# A Novel Intra-body Sensor for Vaginal Temperature Monitoring

**DOI:** 10.3390/s90402797

**Published:** 2009-04-21

**Authors:** Joel J. P. C. Rodrigues, João Caldeira, Binod Vaidya

**Affiliations:** 1 Instituto de Telecomunicações, Department of Informatics, University of Beira Interior, Covilhã, 6200-001, Portugal; 2 University of Beira Interior, Department of Informatics, Covilhã-Portugal; Polytechnic Institute of Castelo Branco, EST, Castelo Branco, 6000-000, Portugal; E-Mail: jcaldeira@est.ipcb.pt; 3 Instituto de Telecomunicações, Covilhã, 6200-001, Portugal; E-Mail: bnvaidya@co.it.pt

**Keywords:** Body Sensor, e-Health, Temperature Monitoring

## Abstract

Over the years some medical studies have tried to better understand the internal behavior of human beings. Many researchers in this domain have been striving to find relationships between intra-vaginal temperature and certain female health conditions, such as ovulation and fertile period since woman’s intra-vaginal temperature is one of the body parameters most preferred in such studies. However, due to lack of a appropriate technology, medical research devoted to studying correlations of such body parameters with certain womans’ body phenomena could not obtain better results. This article presents the design and implementation of a novel intra-body sensor for acquisition and monitoring of intra-vaginal temperatures. This novel intra-body sensor provides data collection that is used for studying the relation between temperature variations and female health conditions, such as anticipation and monitoring of the ovulation period, detection of pregnancy contractions, preterm labor prevention, etc.. The motivation for this work focuses on the development of this new intra-body sensor that will represent a major step in medical technology. The novel sensor was tested and validated on hospitalized women as well as normal healthy women. Finally our medical team has attested to the accuracy, usability and performance of this novel intra-body sensor.

## 1. Introduction

With the advancement of e-health, an increasing number of biomedical sensors are being implanted or be worn in a human body for the monitoring, diagnosis, and treatment of diseases. Nowadays, body sensor systems are very helpful medical diagnosis methods [1–3]. In some of these studies medical researchers have needed to analyze physiological parameters that can only be recorded by electronic sensors [4,5]. These studies often lead to the development of new body sensors that could help in the collection of physiological parameters and provide data for these studies [6–8]. These body sensors could be classified generally in two groups according to their placement: *i*) intra-body sensors, that are placed inside the human body, and *ii*) body sensors that are used outside the human body, most of which operate in contact with skin.

This article presents the development of a novel intra-body sensor that is ideal for precise medical study for the acquisition of female body physiological parameters. According to the studies carried out over the past years, there is a close relationship between certain human body states and different body temperatures (e.g. basal metabolism, muscle contraction, intake of food, and some disease’s symptoms). Even during the day (24 hours) and for the same individual, the body temperature varies by about 0.5 degree Celsius (°C) [9].

The monitoring of the human physiological parameters could help medical staff in correlating temperature readings with some pathologies. Intra-vaginal temperature is one of the female body’s physiological parameters that is the most controlled and useful for monitoring in fertility medical studies [10,11]. For every woman, the characterization of this parameter could help in establishing a pattern for the correlation of intra-vaginal temperature readings and menstrual cycle stage. This could help females to detect their ovulation and fertile periods such that it would be easy to know the best time to get pregnant. Conversely, it could also help females avoid pregnancy if they can know the occurrence of this period.

To the best of our knowledge, none of the existing biosensors can gather continuous, long-term measurements of female intra-vaginal temperatures which are used for estimation and detection of ovulation and fertile periods. All studies based on basal temperature control used a very painful method for the females, in which their basal temperatures have to be taken with a basal body thermometer at specific times (6:30 AM is recommended). Every reading is then used to fill out a fertility chart. With the continuous filling of this chart, females could detect their fertile period by observing the increasing of their basal temperature [10]. Besides the described problems, another debatable issue in this method is the validity of the data gathered. Sometimes the measurement of the temperature, performed by each female, might not be taken in an appropriate way. Most likely, the temperature readings could be wrong on inappropriate handling of the thermometer, thus resulting in incorrect data on the chart.

Our aim was to provide a medical study with a usable device to gather intra-vaginal temperature values during a specific time (e.g. menstrual cycle) defined by the medical staff. This study tried to establish a correlation between intra-vaginal temperature and some stages of the female reproductive system. The data gathered by this device will not only be used to monitor intra-vaginal temperature, but also, to develop studies that could help medical staff gain better knowledge of the relationship between intra-vaginal temperature and some pregnancy issues. Therefore, the main goal of this work was the proposal, creation, testing and validation of a new intra-body sensor for vaginal temperature acquisition and monitoring. The motivation for this work focused on the creation of this new biosensor that will fill a perceived gap in medical technology. This project was conducted jointly with a medical team from the Health Sciences Faculty of the University of Beira Interior, Covilhã, Portugal. This close collaboration is important contribution for medical validation of the accuracy, usability, efficiency, and performance of the system. It also helps to increase the available features and provide continuous improvement of the system to achieve better results. The medical team plans to apply this proposed system in the prevention and detection of the following situations: anticipation and monitoring of the ovulation period (used both as a natural contraception method as well as a estimation of the best fertilization period), detection of pregnancy contractions, effectiveness of some gynaecological therapeutics, preterm-labor prevention, and to support the discovery of new possible contraception methods.

The remainder of this paper is organized as follows. Section 2 presents some studies and projects related to this proposal, while in Section 3, we describe the construction of the new intra-vaginal biosensor. Section 4 focuses on results of first tests and the medical validation of the sensor and Section 5 concludes the paper and presents further research directions.

## 2. Related work

Several devices, developed by research projects, perform monitoring of physiological parameters, such as body temperature. This section presents some of the previous projects for monitoring and analysis of body temperature. The Duofertility Project [12] was already developed as a trail commercial system for monitoring and analysing female body temperatures. This system can detect and calculate both ovulation and fertile periods. Duofertility aims to help couples that want to get pregnant. It measures a female’s body skin temperature by placing a sensor reader under her arm. This sensor is used all the time and records skin temperature measurement every 10 minutes. The sensor device under the arm only collects the skin temperature measurements. For monitoring and analysing the collected measurements, this system uses a remote reader unit. This unit, by on-demand operation, retrieves all collected data from the skin sensor. After the analyses of the received data, the reader unit provides a colour coded signal indicating the potential fertile period.

Another project, ANOM [13], integrated their system with a skin temperature sensor. With this integration the ANOM project aimed to establish a correlation between core body temperature and skin temperature. This attempt was unsuccessful because they concluded that skin temperature could vary with change in environmental conditions, so a correlation between core body temperature and skin temperature could not be established. This failure resulted in the removal of this feature from the ANOM system for medical proposes.

The above-mentioned projects based their temperature studies on measurement and analysis of skin body temperature. As it can be seen in [9] and [14], skin body temperature could be influenced by environmental conditions. This behaviour could lead in poor accuracy and wrong results when skin temperature measurements are used for study.

Another recent research project was conducted with the goal of avoiding the excessive fatigue of American football players by monitoring their intra-body temperature [5]. The research team used a radio pill system developed to monitor the core body temperature in astronauts. This system comprises two modules, a radio pill that must be ingested by a player a couple of hours before the effort, and a wireless reader that is used to monitor the intra-body temperature in real time. If the coach wants to know the core body temperature of a particular player, he can use the reader to check it by pointing the temperature reader at this player. Once this operation is performed the reader shows the real-time temperature for that player and the technical team can proceed in accordance, if needed, resting the player to cool him down. Each radio pill only remains in the body during the digestion time (about 24 to 36 hours). After this period it is eliminated and cannot be used again.

This paper presents a system capable to monitor and analyze the woman’s intra-vaginal temperature in a continuous way. It leads in the development of a thermal sensor that can be placed in the female cervix where it can get a core body temperature. That way the system can get better accuracy in the collected temperature measures leading to more reliable results in this parameter monitoring and analyses. It is also important to mention that this sensor is reusable and very ease to sterilize and wash.

## 3. Intra-vaginal Temperature Sensor

**Figure 1.**
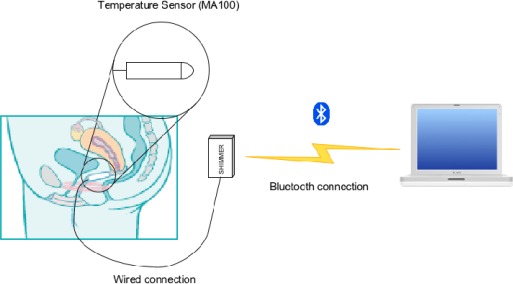
System architecture.

This section presents the design of a system able to gather long-term intra-vaginal temperature measurements. Figure 1 shows the architecture of the developed system.

This architecture comprises three modules: the thermal sensor device, the communication between the device and computer, and the computer application for monitoring and analysing temperature measurements. This article focuses on the first two modules. The following subsections explain the work performed to achieve the final results.

### 3.1. Sensor Design

The proposed work is the conception of a new body-sensor device capable of measuring and gathering long-term female intra-vaginal temperature data. A new biosensor device has been made using the SHIMMER (Sensing Health with Intelligence Modularity, Mobility and Experimental Reusability) platform, which is a wireless sensor platform designed by the Intel Digital Health Group and is used as the processor unit of the biosensor.

This platform consists of following components: an 8 MHz Texas Instruments^™^ MSP430 CPU, a class 2 Bluetooth^®^ radio communication, a 2.4GHz IEEE 802.15.4 Chipcon^™^ wireless transceiver, a 3-Axis Freescale^™^ accelerometer, a MicroSD^™^ slot for up to 2 Gbytes, an integrated Li-Ion battery management and some extension boards (internal and external) that increase the potential of SHIMMER platform with new features and functionalities. All these features are compacted in a very small form factor (2.0 × 4.5 cm) no larger to a thumb [15]. Even though the SHIMMER platform has most of the required features for our design, it does not include a temperature sensor, so one of the challenges was to integrate a temperature sensor into the SHIMMER platform in order to get constant temperature readings. For this integration, we have used one of the available ADC channels in the SHIMMER external board (AnEX board).

For the design of the temperature sensor, we chose the MA100 thermistor, which is a NTC Type MA Biomedical Chip Thermistor developed by GE Industrial Sensing and exclusively used for biomedical applications. Its main features fulfil the requirements of our solution. That means, its sensitivity ranges from 0°C to 50°C, size is 0.762 × 9.52 mm; moreover it is created for biomedical applications. To get more accurate temperature readings, the temperature sensor must be placed inside the female cervix which is an ideal thermal source. For this purpose, the MA100 needs to be placed inside the vagina.

**Figure 2.**
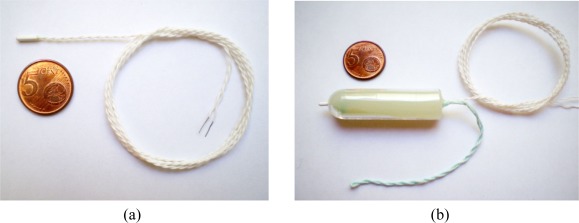
(a) MA100 thermal sensor. (b) MA100 inside enclosure tampon resembles.

Figure 2(a) presents MA100 the thermal sensor used in this system. However, since the MA100 is very flexible, it is very difficult to place it at the right position inside the vagina, so for encapsulating the MA100 unit, a tampon-like specialized enclosure was designed which is not only anatomically comfortable for women but also robust and easy way to use. Figure 2(b) shows the MA100 with the enclosure. Correct placement of the temperature sensor, with this configuration, is trivial for women and should not cause any problems while in use, as using this intra-body sensor is like using a traditional tampon. Hence, to place the enclosed MA100 inside the vagina, we follow a similar procedure as for any traditional tampon. Furthermore, in order to achieve a reusable intra-body sensor, we have considered that it should be easily sterilized and washable.

### 3.2. Operating Principles

**Figure 3.**
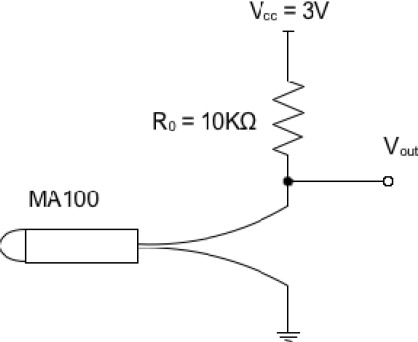
Electronic circuit for thermal sensor

The developed intra-body sensor comprises two parts: a thermal temperature sensor (MA100) with an enclosure and a processor unit (SHIMMER platform). The temperature sensor (MA100) actually is the only part of the system that is placed inside female’s body. To achieve accurate temperature readings, the MA100 is placed in the cervix. The processor unit (SHIMMER) is placed outside of the woman body, because of bulky size, and could be placed anywhere if the cable is long enough (∼ 80 centimeters). For example, it may be easily placed inside a pocket, attached to a belt etc.. The temperature sensor (MA100) measures the voltage values and sends them to SHIMMER using a wired flexible connection. Each voltage value has a linear correspondence to one temperature value, according to the analysis of the supporting electronic circuit. This analysis gives us the real-time temperature measured by the temperature sensor. Figure 3 presents the electronic circuit developed for the integration of the MA100 thermal sensor in the system. The analysis of this circuit is translated by equation (1) where *T* corresponds to the temperature measurement, in degree Celsius (°C). In this equation, *V*_0°*C*_ corresponds to voltage measurement (in milli-volts) at 0°C temperature; *V_out_* corresponds to the measured voltage at the terminals of the MA100 thermistor; and the constant value 30.04 corresponds to the variation of increasing temperature value 1 degree Celsius (°C), in milli-volts.

(1) $$T=\frac{V_{0\;{}^{\circ}C}\;[\mathrm{mV}]\;-\;V_{\mathrm{out}}\;[\mathrm{mV}]}{30.04\;[mV/{}^{\circ}C]}\;[{}^{\circ}C]$$

**Figure 4.**
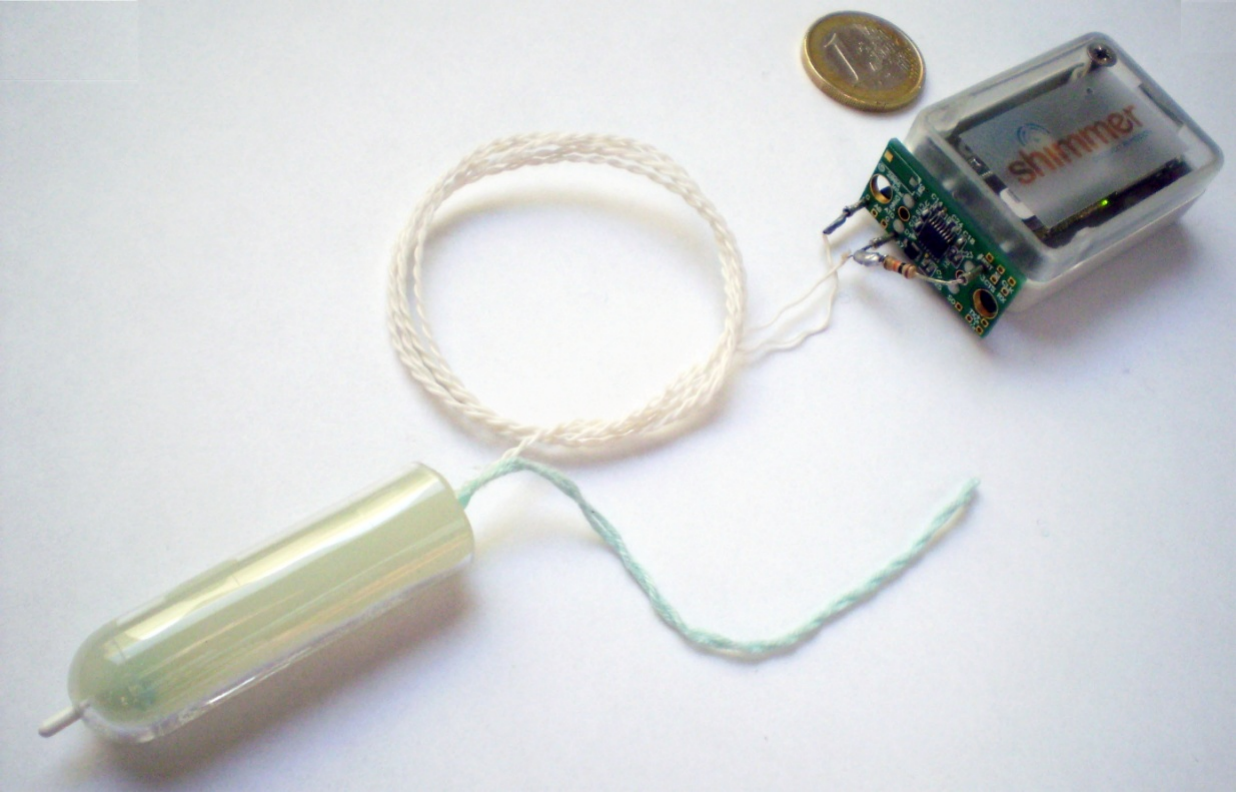
Developed system prototype.

In Figure 4, the developed intra-body sensor prototype to gather long-term intra-vaginal temperature measurements is presented.

To gather the temperature measurements of the sensor, SHIMMER provides a microSD slot. This feature avoids the need for constant communication with a personal computer in order to receive and store each temperature measurement. In this way, the system can work in a stand-alone mode collecting temperature measurements for a long period. Computer communication is needed to operate SHIMMER. This communication is also used to retrieve all temperature measurements gathered in the SHIMMER’s microSD card to the computer for the analysis.

The communication between SHIMMER and computer is performed by a Bluetooth connection. The computer sends operating commands to SHIMMER, which in turn, sends information and data to computer. SHIMMER always waits for computer commands over the Bluetooth connection. Once a command is received then it proceeds accordingly. The operating commands available in SHIMMER are the following:

- *Start*: when SHIMMER receives this command, it starts collecting temperature measurements in the microSD card. If a Bluetooth connection continues available temperature measurements are also delivered to computer for real-time monitoring and analyses. If no connection is available, temperature measurements are only written in microSD card. That way, SHIMMER prevents unnecessary use of Bluetooth connection and allows increasing the battery lifetime.
- *Stop*: this command stops the collection of new temperature measurements.
- *Get*: this command performs the transmission of all temperature measurements gathered in microSD card to the computer application for further study and analysis.

**Figure 5.**
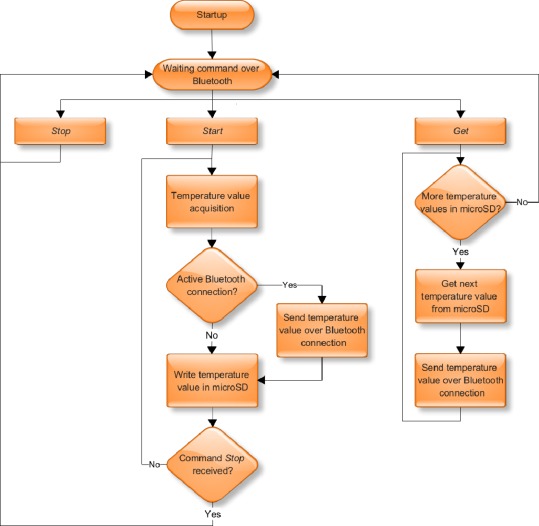
SHIMMER firmware diagram.

Figure 5 presents the firmware diagram implemented in SHIMMER. This firmware provides all the above-mentioned features.

The analysis of temperature measurements is performed in off-line mode, so to ensure good results, it is vitally important to know the exact time when each temperature measurement is taken. SHIMMER only has a local time clock, which starts on SHIMMER’s start up. This clock can’t give the real global time clock. To associate each measurement with the right global time clock instant, when a *start* command is send to SHIMMER, computer also sends its clock and date time (assuming computer clock is global clock synchronized). This information is then used by SHIMMER’s firmware as an offset to local time clock in order to calculate the exact global time clock for every instant of temperature measurement.

## 4. Experimental Results

In order to validate the comfort, usability and accuracy of the proposed system, the medical team performed several tests in the hospital. These tests were only possible with the collaboration of the hospitalized women who agreed to participate in the tests. Twelve women were monitored with the intra-vaginal sensors for about sixty minutes, under medical supervision. In all the tests, an interval of one second was used for each new temperature measurement. This gave us a total of 3,600 measurements for each test. All these values were then analysed in off-line mode by the medical team. They compared it with temperature measurements performed at the same time for each woman under the arm and under the tongue. In this case, the temperature measurements were gathered in a traditional way by using standard digital thermometers. After analysing the temperature measurements acquired using both methods for each test (the traditional way and with the intra-vaginal sensor), the medical team concluded that the results obtained from the intra-vaginal sensor gave accurate results. We also verified the comfort of the thermal sensor during the test. At the end, each woman was asked to report any complications in using the intra-vaginal sensor. None of them report any problem in using the intra-vaginal sensor, and some even said, “It is just like using a tampon”. Thus the newly designed intra-vaginal sensor is comfortable as well as very easy to use.

Additional tests were performed for eight women during their normal daily activities. These tests result from the monitoring of these women in different periods of the day. Each test lasted about two to three hours. The validation of the performed tests was possible according to temperature measurement patterns previously established for these women. These patterns were defined using the same method described above (measuring the temperature under the arm and under the tongue). The medical team confirmed the accuracy of the temperature measurements from the intra-vaginal sensor when compared to earlier patterns.

**Figure 6.**
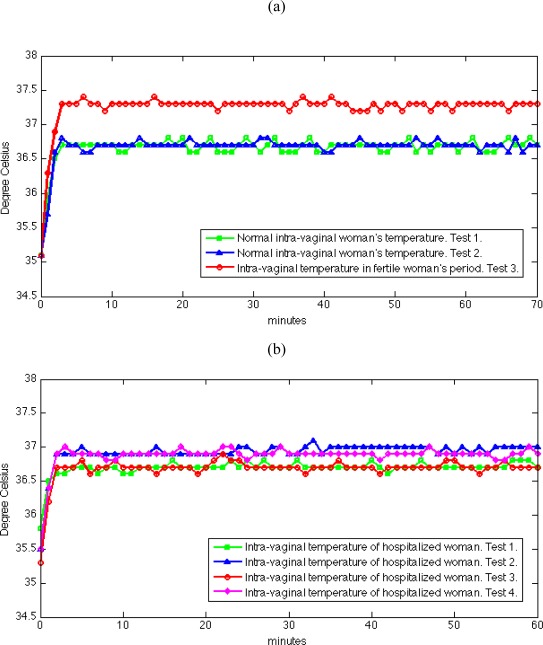
(a) Samples of performed tests in day life woman. (b) Samples of performed tests in hospitalized women.

Figure 6 presents a sample of the tests performed in hospitalized and normal day life women. In Figure 6(a), the results shown in the graph correspond to three tests performed on the same woman on different days. Each graph can be divided in two parts. The first part, from zero minute to the second minute corresponds to the warm-up of the sensor to reach the vagina’s internal temperature. The second part, from third minute onwards represents the actual temperature inside the woman’s vagina. It can be seen that two curves with squares and with triangles differ in average of about 0.5 °C from the curve with circles. This difference corresponds to the observation of normal body temperature (curve with squares and curve with triangles) and fertile period temperature (curve with circles) for this woman. As we expected, different stages in a woman’s body reflect variations in intra-vaginal temperature. The correlation of these variations and the occurrence of some situations in woman’s body could help medical staff achieve in a better understanding of the female reproductive system. This system could even help women that want to monitor their self in detection of fertile and ovulation period (used both as a natural contraception method or, conversely, as an estimation of the best fertilization period). Furthermore, women could use this system only to better understand their intra-vaginal temperature regulation.

Figure 6(b) presents the result of four tests performed in the hospital. These tests correspond to four different women. To construct the graph, the above-mentioned procedures can be applied into these tests too. From third minute onwards, as above, the lines are the actual intra-vaginal temperature measurements for these four women. The conclusion of these tests is that each woman has her own intra-vaginal temperature pattern. Therefore, it is necessary to define these patterns. This system could be part of a health-care management system, it that it can store the intra-vaginal temperature measurements obtained from each woman, which can be applied for further use.

The great results achieved in these first tests by the medical team encouraged them to continue the development of new tests in order to achieve better results and improve the available features in the system. The medical team also wants to study the possibility of new applications of the sensor in other medical applications by finding new correlations between human body phenomena and variations of core body temperatures.

## 5. Conclusions and Future Work

Body-sensors are constantly used in medical procedure nowadays. They help in patient’s healthcare by monitoring and analysing human body parameters. This article presents the development of a novel intra-vaginal sensor for monitoring and analysing core body temperatures. It can help medical staff and women in some reproductive system issues that can be detected by their correlation with intra-vaginal temperature. Some of these issues are related to the anticipation and monitoring of the ovulation period, detection of pregnancy contractions, effectiveness of some gynaecology therapeutics, preterm labor prevention, and supporting the discovery of new possible contraception methods. The developed system has been already tested in practical situations (twenty tests were performed) in a hospital and this led to its validation by a medical team. Other tests were performed during the normal day life of eight women. These tests confirmed the versatility and the easy use of the developed intra-vaginal sensor. The medical team that assisted with this project also validated the results of these tests. In the future, our main concern is the miniaturization of the actual intra-vaginal sensor such that it (MA100 together with SHIMMER) can be easily placed inside female cervix. This aim will definitely provide more flexibility and provide the ease and comfort on using this sensor.
